# Solute Carrier Nucleoside Transporters in Hematopoiesis and Hematological Drug Toxicities: A Perspective

**DOI:** 10.3390/cancers14133113

**Published:** 2022-06-25

**Authors:** Syed Saqib Ali, Ruchika Raj, Tejinder Kaur, Brenna Weadick, Debasis Nayak, Minnsung No, Jane Protos, Hannah Odom, Kajal Desai, Avinash K. Persaud, Joanne Wang, Rajgopal Govindarajan

**Affiliations:** 1 Division of Pharmaceutics and Pharmacology, College of Pharmacy, The Ohio State University, Columbus, OH 43210, USA; ali.885@osu.edu (S.S.A.); rraj@uaschools.org (R.R.); kaur.301@osu.edu (T.K.); hepp.100@osu.edu (B.W.); nayak.83@osu.edu (D.N.); no.20@osu.edu (M.N.); protos.3@osu.edu (J.P.); hannah@hannahodom.com (H.O.); desai.399@osu.edu (K.D.); persaud.19@osu.edu (A.K.P.); 2 Department of Pharmaceutics, College of Pharmacy, University of Washington, Seattle, WA 98195, USA; jowang@uw.edu; 3 Translational Therapeutics, The Ohio State University Comprehensive Cancer Center, Columbus, OH 43210, USA

**Keywords:** nucleoside, anemia, transporter, hematological, drug, myelosuppression, toxicity

## Abstract

**Simple Summary:**

Anticancer nucleoside analogs are promising treatments that often result in damaging toxicities and therefore ineffective treatment. Mechanisms of this are not well-researched, but cellular nucleoside transport research in mice might provide additional insight given transport’s role in mammalian hematopoiesis. Cellular nucleoside transport is a notable component of mammalian hematopoiesis due to how mutations within it relate to hematological abnormities. This review encompasses nucleoside transporters, focusing on their inherent properties, hematopoietic role, and their interplay in nucleoside drug treatment side effects. We then propose potential mechanisms to explain nucleoside transport involvement in blood disorders. Finally, we point out and advocate for future research areas that would improve therapeutic outcomes for patients taking nucleoside analog therapies.

**Abstract:**

Anticancer nucleoside analogs produce adverse, and at times, dose-limiting hematological toxicities that can compromise treatment efficacy, yet the mechanisms of such toxicities are poorly understood. Recently, cellular nucleoside transport has been implicated in normal blood cell formation with studies from nucleoside transporter-deficient mice providing additional insights into the regulation of mammalian hematopoiesis. Furthermore, several idiopathic human genetic disorders have revealed nucleoside transport as an important component of mammalian hematopoiesis because mutations in individual nucleoside transporter genes are linked to various hematological abnormalities, including anemia. Here, we review recent developments in nucleoside transporters, including their transport characteristics, their role in the regulation of hematopoiesis, and their potential involvement in the occurrence of adverse hematological side effects due to nucleoside drug treatment. Furthermore, we discuss the putative mechanisms by which aberrant nucleoside transport may contribute to hematological abnormalities and identify the knowledge gaps where future research may positively impact treatment outcomes for patients undergoing various nucleoside analog therapies.

## 1. Introduction

In cell physiology, nucleosides play vital roles as precursors for cellular macromolecules including ribonucleotides and deoxyribonucleotides. They are also involved in the synthesis of nucleotide cofactors, such as S-adenosylmethionine and nicotinamide adenine dinucleotide. Moreover, they act as ligands (e.g., adenosine) and take part in cellular signaling by purinergic receptors. Naturally occurring nucleosides are subdivided into purine nucleosides (adenosine, guanosine, and inosine) and pyrimidine nucleosides (uridine, cytidine, and thymidine). With the molecular cloning and expression of cDNA, in addition to the characterization of solute transport in orthologous systems, two families of nucleoside transporting proteins have emerged in humans: high-capacity, low-affinity equilibrative nucleoside transporters (ENTs) and low-capacity, high-affinity concentrative nucleoside transporters (CNTs) [1]. The human ENT family can be divided into four subtypes hENT1, hENT2, hENT3, and hENT4, while the hCNT family can be classified as hCNT1, hCNT2, and hCNT3 [1]. The best-characterized function of plasma membrane-bound ENTs (i.e., ENT1 and ENT2) is that they can accept both purine and pyrimidine nucleosides as cargos to equilibrate nucleosides across the cellular membrane. Additionally, hENT2 is amenable for the transport of specific nucleobases (e.g., hypoxanthine). A hint regarding the primary function of hENT3 can be inferred from its predominant localization on organelle membranes, regulating the intracellular compartmentalization of nucleosides in lysosomes and mitochondria at a low pH [1]. hENT4 only transports adenosine at a low pH [2,3], and it has been reclassified as a plasma membrane monoamine transporter because of its predominant acceptance of monoamines as solutes. Nitrobenzylmercaptopurine ribonucleoside (NBMPR) is a relatively specific transport inhibitor that can be used to differentiate hENT1 (equilibrative sensitive; es) from hENT2 transport (equilibrative insensitive; ei) activities [4,5,6]. hENT1 and hENT2 are also inhibited by nonnucleoside inhibitors that are vasodilator drugs, such as dilazep and dipyridamole [7]. Unlike ENTs, the uptake of nucleosides by CNTs is unidirectional as it is coupled with the influx of sodium ions, although CNT3 can accept other protons in place of sodium ions. The sodium/nucleoside coupling ratio is different for CNTs, which is 1:1 for CNT1 and CNT2 and 2:1 for CNT3. Uridine is the only nucleoside that can be transported by all CNT subtypes, which are otherwise very selective for substrates [8]. For instance, CNT1 is selective for the transport of pyrimidine nucleosides and CNT2 for the transport of purine nucleosides, while CNT3 conducts the transport of both purine and pyrimidine nucleosides [9,10,11,12,13]. Currently, there are no specific inhibitors available for CNTs, although research is underway for the development of CNT inhibitors. Table 1 summarizes the endogenous nucleoside transport characteristics of ENTs and CNTs.

The physiological, pharmacological, and toxicological roles of nucleoside transporters have been elegantly reviewed in multiple articles in recent years [1,8,9,10,11,12,13,14,38,39,40,41]. This paper discusses the new advances of nucleoside transporters in hematological toxicities of anticancer nucleoside drugs. In addition, the paper summarizes the newly emerging role of nucleoside transporters in mammalian hematopoiesis and discusses potential future research opportunities in this area.

## 2. Cellular Transport of Anticancer Nucleoside Analogs

**Figure 1:**
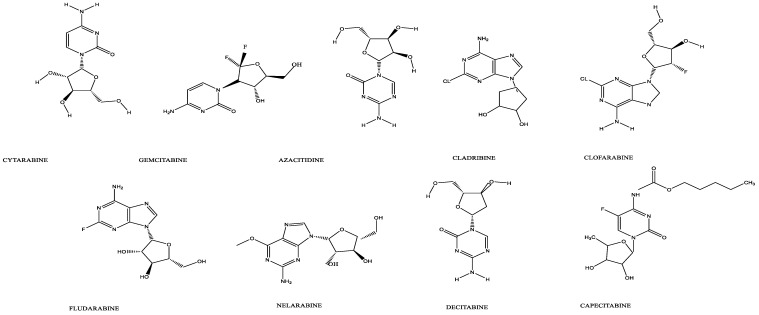
Structure of anticancer nucleoside analogs.

The most clinically relevant function of NTs is the cellular uptake of pyrimidine and purine nucleoside analog drugs used as therapeutic agents for the treatment of many disorders, including cancer. Nucleoside transporters play an indispensable role in antimetabolite drug sensitivity by mediating the uptake of these otherwise highly hydrophilic, poorly membrane-permeable, modified nucleosides that have a higher affinity for the aqueous phase than the lipid phase (a negative value for LogP). To date, nearly a dozen anticancer nucleoside analogs have been approved by the US FDA, which partially or fully depend on nucleoside transporters for cellular uptake and therefore their efficacy [Figure 1] [42,43]. Whereas the first-generation nucleoside analog drugs are very hydrophilic and highly dependent on nucleoside transporters for drug activity, the subsequent-generation drugs are designed to be less dependent, if not completely independent, on nucleoside transporters [42,43]. Nevertheless, normal cell types with high proliferation rates, such as regenerative blood cells, easily succumb to damage by nucleoside analog drugs that are intended to target proliferative and invasive cancer cells. It is noteworthy that numerous anticancer drugs produce a variety of hematological toxicities that remain a significant concern for both patients undergoing treatments for cancers and clinicians treating these conditions [42]. Several examples are described in the following section to illustrate this phenomenon.

## 3. Hematological Toxicities of Anticancer Nucleoside Drugs

Cytarabine is a modified pyrimidine nucleoside in which cytosine is attached to D-arabinofuranose through a beta-N(1)-glycosidic bond. It is mainly used in the treatment of acute leukemias, especially acute nonlymphoblastic leukemia, acute myelogenous leukemia (AML), and non-Hodgkin’s lymphoma. Sometimes, it is used in combination with other chemotherapeutic agents that are used to treat other malignancies, such as multiple myeloma, but it is also favorable as a single agent. Cytarabine enters cells primarily through a hENT1-mediated process [44]. After entry, it is phosphorylated by deoxycytidine kinase (dCK) to the 5′-derivative araCMP and subsequently by nucleotide kinases to araCTP. Cytarabine cytotoxicity results from the inhibition of DNA polymerase α and from the incorporation of araCTP into DNA in place of deoxycytidine triphosphate (dCTP). When araCTP is incorporated into DNA, it prevents DNA chain elongation, which results in the blockage of DNA synthesis. Cytarabine is clearly a bone marrow suppressant, as this milieu is host to the highly proliferative process of blood cell formation. Therefore, thrombocytopenia, neutropenia, and reduced reticulocytes are reported after the administration of cytarabine [44]. The morphology of bone marrow and peripheral smears at the cellular level can be significantly changed after administration of cytarabine, and the gravity of this drug toxicity is dose- and time-dependent. Myeloid leukemia patients reported a range of frequencies of anemia, thrombocytopenia, and neutropenia at dosages ranging from 20 mg to 50 g/m^2^ day (Table 2) [45,46,47,48,49,50,51].

Gemcitabine is a similar drug to cytarabine but is used to treat various solid tumor malignancies. Chemically, it is a 2’-deoxycytidine with geminal fluoro substituents in the 2’-position. As a single agent, it is approved for the treatment of nonresectable pancreatic cancer [144], although it is currently used more frequently in combination with Abraxane (nab-paclitaxel). Gemcitabine is also used in combination with cisplatin to treat bladder cancer [145]. Like cytarabine, gemcitabine is also transported by nucleoside transporters. In fact, many nucleoside transporters belonging to both the ENT and CNT families play a role in the transport of gemcitabine inside the cell. Of many transporters, hENT1, hENT2, hCNT1, and hCNT3 are functionally characterized for the uptake of gemcitabine inside the cell [11], with hENT1 and hCNT1 reported as the most effective transporters to transport gemcitabine into cells. When gemcitabine enters cells, it is phosphorylated into its monophosphate form by dCK (dFd-CMP), and pyrimidine nucleotide kinases then help convert it into its active triphosphate form (dFd-CTP) [146]. Cytidine deaminase is capable of inactivating gemcitabine by deamination [147,148]. Gemcitabine can prevent DNA repair and chain termination as dFd-CTP is incorporated into the growing DNA strand, in addition to allowing the addition of natural nucleosides [148]. In comparison, between cytarabine and gemcitabine, the latter accumulates in a prolonged fashion, as it can inhibit ribonucleotide reductase and deoxycytidine monophosphate deaminase for a longer period [149]. Similarly, gemcitabine is a better substrate for nucleoside transporters than cytarabine given that it phosphorylates rapidly and removes itself more slowly from the cells; therefore, its active metabolites have a higher retention rate than those of cytarabine. However, gemcitabine also has a strong connection with the induction of hemolytic uremic syndrome (HUS), which is rare but can be fatal. This syndrome is characterized by microangiopathic hemolytic anemia and thrombocytopenia [150]. Although it is unclear at this time why certain patients experience HUS, it can be noted that multiple studies demonstrate widely varied expression of hENT1 in solid tumors, which impacts drug uptake and availability [150]. Patients suffering from pancreatic cancer and taking gemcitabine as a chemotherapeutic agent can suffer from anemia at a dose of near 1000 mg/m^2^ [150]. Gemcitabine is also associated with various other hematological toxicities, such as neutropenia, when given in combination with different chemotherapeutic agents, such as cisplatin and docetaxel. The combination of gemcitabine and docetaxel has been found to be an effective candidate for the treatment of non-small cell lung cancer (NSCLC), although the use of this combination has dose-limiting toxicity. Although studies show that docetaxel (30 mg/m^2^) and gemcitabine (700 mg/m^2^) used in this range do not have toxicity, when the dose of this combination exceeds this limit, patients may suffer from toxicities, particularly neutropenia. Other studies show that when the dose of gemcitabine increases up to 800 mg/m^2^, patients may begin to suffer from neutropenia [151]. Gemcitabine is also associated with lymphocyte toxicity in patients treated for aggressive pancreatic ductal adenocarcinoma [151]. Gemcitabine also has a dose-limiting effect on the lymphocyte count (lymphopenia) when the dose increases above 800 mg/m^2^ in patients [151,152] (Table 2).

Azacitidine, another analog of cytidine, is used for the treatment of various hematological malignancies. In fact, azacitidine was the first DNA hypomethylating agent approved by the FDA for the treatment of myelodysplastic syndromes (MDS). Azacitidine is used for the treatment of high-risk MDS and AML. DNA hypomethylation is a major reason for the antineoplastic activity of azacitidine and its cytotoxic effect on abnormal hematopoietic cells in the bone marrow. Both hCNTs (hCNT1 and hCNT3) and hENTs (hENT1 and hENT2) are involved in the cellular uptake of azacitidine. The uptake of azacytidine by hCNTs and hENTs has been demonstrated in cell-based studies and Xenopus oocytes exogenously expressing human transporters [31,153,154]. It is incorporated into both RNA and DNA, but to a lesser extent into the latter. Incorporation into RNA disrupts normal RNA function and impairs tRNA cytosine-5-methyltransferase activity. This incorporation leads to cytotoxicity by impeding the synthesis of nucleic acids and proteins mainly by affecting rapidly dividing cells. In patients with AML, azacitidine is a proven cytotoxic agent at doses of 100 to 750 mg/m^2^/day [155]. It has time- and dose-dependent toxicity, and prolonged exposure to this nucleoside analog allows it to enter DNA and induce apoptosis [156,157]. Clinically observed hematological toxicities include neutropenia, thrombocytopenia, and anemia. The efficacy of azacitidine was evaluated in patients with high-risk MDS. In a study evaluating toxicities, azacitidine was taken either alone or in combination with enasidenib. The most common side effects observed were gastrointestinal and hematological perturbations. Sixty-two patients received a combination of azacitidine and enasidenib, while 32 patients received azacitidine alone. Those who received the combination were at a higher risk of developing neutropenia, thrombocytopenia, and anemia than those receiving azacitidine alone. The risks of developing neutropenia, anemia, and thrombocytopenia were 25%, 22%, and 19%, respectively, in patients who were taking azacitidine alone [158]. In another study, clinicians used azacitidine to treat MDS and AML, and reported a similar side effect profile for this nucleoside derivative. A total of 236 patients were taken into consideration and were given 300 mg for 14 days to treat AML. The frequencies of thrombocytopenia, anemia, and neutropenia were 33%, 20%, and 44%, respectively [159]. The toxic effect of azacitidine was also evaluated in another cohort of MDS patients. A total of 107 patients were taken into consideration: 300 mg/day was given to them for 21 days and the side effects were analyzed. Of these 107 patients, 20 were affected by anemia, 31 by thrombocytopenia, and 50 by neutropenia [159,160]. The frequency ranges of various hematological toxicities observed in various studies are shown in Table 2.

Cladribine (2-chloro-2′-deoxyadenosine) is an analog of deoxyadenosine. Cladribine is effective in treating hairy cell leukemia, which is a B-cell lymphoproliferative disease. This purine analog can result in up to 90% complete remission even after one course of treatment [161]. However, like other nucleoside analogs, such as cytarabine and gemcitabine, it produces cytotoxicity after these analogs become phosphorylated [162,163,164]. Both CNTs and ENTs are involved in the transport of this drug into the cell. Along with CNTs and ENTs, breast cancer resistance protein (BCRP) is also involved in the transport of cladribine, but the transporters that play a major role include ENT1, ENT2, and ENT4. Unlike ENTs, CNT2 and CNT3 are involved in the transport of cladribine with low affinities [165]. Deoxycytidine kinase is the major enzyme that phosphorylates cladribine, which accumulates as chlorodeoxyadenosine triphosphate [166,167]. This enzyme has high activity in lymphocytes along with low 5-nucleotidase, which unfortunately shows high selectivity for these cells [168]. Other studies showed that cladribine achieved its efficacy after a single 7-day course of administration of the drug, with an acceptable safety profile. However, circulating mononuclear cells, such as lymphocytes and monocytes, were affected by cladribine toxicity [163]. The toxicity of cladribine, like that of other nucleoside analogs, is also dose-dependent. At lower doses, cladribine causes monocytopenia and lymphopenia; at higher doses, it has been predicted to cause thrombocytopenia, neutropenia, and anemia [169,170]. The effectiveness of this drug and its toxicity measured in other studies sheds additional light on cladribine toxicity. In a study conducted by Robak et al., patients were administered a dose of 0.12 mg/kg daily for 5 consecutive days. In this study, 113 patients who had taken the indicated dose were considered. Of these, 76 were previously treated with chlorambucil and prednisone, and 46 were untreated. The frequency of thrombocytopenia in those patients who were untreated was found to be 32.7%, whereas that in those who were previously treated was 35.8%, suggesting that nucleoside analogs are a predominant cause of thrombocytopenia in this scenario [135]. Other hematological toxicities, such as anemia and neutropenia, also occurred in the patients. The frequency of neutropenia was found to be 11.5%, and that of anemia was 8.8% (Table 2).

Clofarabine is another purine nucleoside analog that is considered a better version of cladribine and fludarabine because it contains the best qualities of both drugs but has reduced toxicity. hENT1, hENT2, and hCNT3 are the three transporters that allow clofarabine to enter cancer cells [171,172]. The inhibition of ribonucleotide reductase is the major mechanism through which it functions; however, it also induces apoptosis of cancer cells [173,174,175]. In several comparable studies, the efficacy of clofarabine was measured in AML patients. Megan and colleagues [84] analyzed 22 patients who were 60 years or older. Clofarabine dose ranged from 1 to 6 mg and was given to patients once daily for 14 or 21 days of a 28-day cycle, and the adverse effects of clofarabine were evaluated. Thrombocytopenia and neutropenia were observed, and found in a 50:50 ratio among patients. Another study performed by the Abramson group studied the toxicity and efficacy of clofarabine in patients with relapsed/refractory non-Hodgkin’s lymphoma. The primary toxicities were like those in the above study and were characterized by thrombocytopenia, neutropenia, and anemia. In another study [176], a total of 30 patients were taken into consideration, and doses ranging from 1 to 4 mg were examined. The common toxicities were thrombocytopenia (30%), neutropenia (60%), anemia (63%), and fatigue (27%). Together, the toxicity data reported for clofarabine consistently point to thrombocytopenia and neutropenia (Table 2).

Fludarabine is a fluorinated nucleoside analog with antineoplastic activity and has been used to treat CLL over the last two decades. It is also used to treat other malignancies, such as non-Hodgkin’s lymphoma. Over the years, its applicability in cancer treatment has become limited because it is known to produce serious toxicities. The major toxicity associated with fludarabine is myelosuppression and the development of secondary malignancies [177]. hENT1 and hCNT3 are transporters involved in the cellular uptake of this drug [172]. When fludarabine enters the cytoplasm, it is first phosphorylated to monophosphate (F-araAMP) by dCK, after which it is converted to F-araATP by subsequent phosphorylation [178]. Enzymes that are involved in the synthesis of nucleoside and DNA replication, such as DNA polymerase [148,179], DNA primase [180], ribonucleotide reductase [179], and DNA ligase I [181], are inhibited by F-araATP. In nonreplicating cells, cytotoxicity is mainly due to the obstruction of cellular DNA repair processes [182]. In a study evaluating 172 patients, myelosuppression was predominantly noted as a significant adverse drug reaction. It was found that 12.7% of the patients suffered from thrombocytopenia, and anemia was found in 11.6% of patients [178]. Numerous studies on chronic lymphocytic leukemia have indicated that 10 to 25% of patients suffered from anemia [61,62,63], 4 to 42% from thrombocytopenia [93,94,95,96,97], and 10% from neutropenia, after administration of fludarabine at a dosage level of 25–30 mg/m^2^/day [93,129,130]. A few reports have revealed the development of eosinophilia in non-Hodgkin’s lymphoma and chronic lymphocytic leukemia patients with a peak eosinophil count of 7.9 × 10^9^/L [140,141,142] (Table 2).

Nelarabine is also a purine nucleoside analog, a prodrug of arabinosylguanine nucleotide triphosphate (araGTP) that causes inhibition of DNA replication and cytotoxicity. [3]. It is primarily indicated for the treatment of ALL and T-cell lymphoblastic lymphoma that have not responded to other chemotherapy regimens. Nucleoside transporters that are involved in the transport of this drug belong to the ENT family, and ENT1 and ENT2 are reported to transport nelarabine effectively into cells [67]. Once inside cells, adenosine deaminase demethylates nelarabine into its active form, araG, which subsequently phosphorylates to araGTP. The araGTP form of the drug is incorporated into the DNA and inhibits its synthesis, which leads to DNA fragmentation and cell death [67]. In T-ALL patients, nelarabine was administered at 1.5 mg/day on alternate days for a week, and the adverse effects were analyzed. The primary toxicity that occurred in these patients was hematological toxicity. The frequency of anemia was found to be approximately 37%; thrombocytopenia—8 to 23% [101,102,103,104]; lymphopenia—79% [103,120,121]; and neutropenia—37 to 46% [67,102,132] (Table 2).

Table 1 summarizes the nucleoside drug transport characteristics of ENTs and CNTs.

Table 2 summarizes examples of the clinically observed hematological toxicities of commonly used anticancer agents and frequency ranges of toxicity observed.

## 4. Understanding Hematopoiesis for Interpreting Hematotoxicity

**Figure 2:**
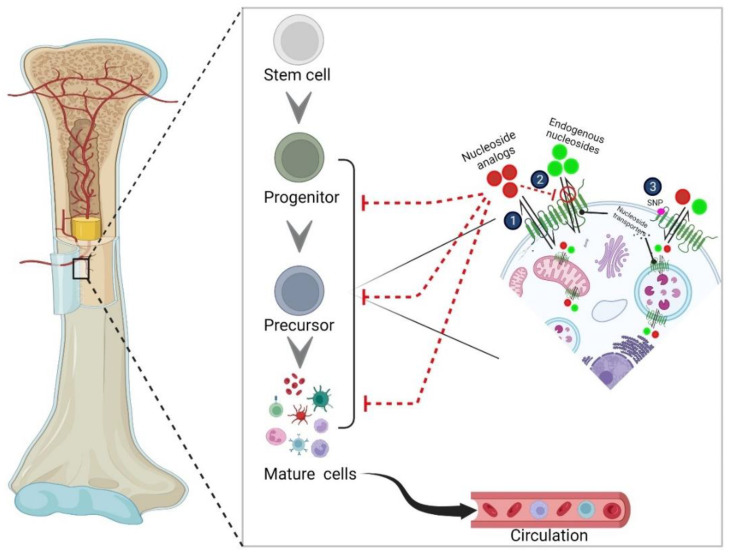
**Putative mechanisms of transporter involvement in nucleoside analog toxicity.** 1. Direct entry of nucleoside analogs into hematopoietic cells via nucleoside transporters. 2. Impairment of endogenous nucleoside transport in hematopoietic cells. 3. Single nucleotide polymorphisms in nucleoside transporters. Arrowheads indicate the differentiation path of hematopoietic stem cells. Dotted red lines indicate blockage or impairment. Image created by BioRender.com.

Addressing hematotoxicity using nucleoside analog drugs requires an understanding of hematopoiesis, mechanisms of hematotoxicity, and hematopoietic tissue responses to nucleoside analog drug insults. The human body’s ability to regenerate the blood supply is paramount to survival. Hematopoiesis, the formation of all blood cellular components [183], is executed throughout life, as the body attempts to replenish the blood supply after injuries, infections, transplantations, and other losses of blood. It is estimated that humans produce approximately one trillion blood cells per day through hematopoiesis. Due to the need for a constant and lifelong blood supply, hematopoiesis is a highly regulated process that includes many populations of specialized cells. These specialized cells come from hematopoietic stem cells (HSCs), which are predominantly found in the bone marrow [Figure 2] [184]. HSCs vary both structurally and functionally depending on their life stage. Adult HSCs are multipotent, meaning that their differentiation is limited to their tissue of origin, specifically blood cells [185]. They focus on the maintenance and repair of blood cells, ranging from erythrocytes to leukocytes to platelets [183]. This function makes adult HSCs critical to the process of hematopoiesis. To execute their specialized maintenance and repair functions, HSCs give rise to descendants known as hematopoietic progenitor cells (HPCs), which can either self-renew or further differentiate into specific blood cells [22]. HSCs inhabit a highly regulated environment, often termed “the niche”, referring to the microenvironment in which the cells reside [186]. The niche dictates signaling to HSCs that then decide when the cells should proliferate, self-renew, differentiate, or migrate [186]. Niche regulatory signals vary in their forms, including cell-bound or secreted factors, in addition to cues from other HSCs or nonhematopoietic cell types, including mesenchymal and endothelial stem cells. When investigating diseases such as cancer or blood autoimmune disorders, the importance of HSCs and HPCs becomes strikingly evident. HSCs can increase proliferation if required to replenish the progenitor pool when a higher rate of functional cell generation is required. This provides a mechanism for the potential treatment of blood cancers, autoimmune disorders, cardiac diseases, genetic disorders, and many other illnesses [187]. However, the myelotoxicity that arises from most anticancer drugs results in the decrease in the production of rapidly dividing cells, such as blood cell progenitors and precursors, known as hematological toxicity [188]. Although numerous factors can impact hematopoietic homeostasis and help restore hematopoiesis during and after chemotherapy, interestingly, emerging studies are uncovering a new role for nucleoside transporters in the regulation of basal hematopoiesis and maintenance of hematopoietic progenitor and mature blood cell populations. The subsequent sections discuss these findings, which may have implications for the occurrence and treatment of hematological toxicities mediated by anticancer nucleoside analog drugs.

## 5. A Role for Nucleoside Transporters in Mammalian Hematopoiesis

Using homologous recombination to disrupt nearly 500 genes encoding secretion and membrane proteins, it was discovered that the third member in the ENT family (i.e., ENT3) is a crucial player in the development of RBCs in mice (Figure 1; top) [189]. Subsequent studies identified mice deficient for ENT3 that spontaneously developed anemia beginning at approximately 8 weeks, reaching severe mortality rates at approximately 18–20 weeks due to bone marrow failure [190,191]. Interestingly, an increasing number of human genetic disorders are being attributed to mutations in the human SLC29A3 gene (encodes ENT3) with anemia, bone marrow failure, and hepatosplenomegaly as common accompanying features. These disorders exhibit a heterogeneous manifestation of symptoms, such as histiocytosis, hypertrichosis, hyperpigmentation, pancreatitis, and autoimmunity, and therefore are reported under various diagnostic terms, such as H syndrome, PHID syndrome, RDD, SHML, familial histiocytosis, familial plasmacytosis, and POEMS in children. Even though such disorders can arise from SLC29A3 mutations (>100 reports documenting nearly 30 distinct mutations), the biochemical basis of these disorders remains unclear. Numerous treatments, including surgery, corticosteroids, insulin, tocilizumab, IL-6 blockade, and multiple other immunomodulatory therapies, have been attempted to treat patients with SLC29A3 disorders; however, none of them produced dramatic improvements in patient conditions, suggesting that hENT3 transport is indispensable to normal blood cell formation [192,193,194,195]. In some patients with severe anemia, blood transfusions were tried. In other patients who did not respond well to multiple blood transfusions alone, hematological stem cell transplantation or stem cell transplantation were contemplated; however, the latter approaches remain only in investigational stages.

As mentioned earlier, the solute carrier 29 (SLC29) gene family encodes three equilibrative nucleoside transporters (ENTs; ENT1 to ENT3) essential for the membrane translocation of nucleosides required for salvage DNA synthesis [1]. Whereas ENT1 and ENT2 are localized at the plasma membrane and conduct nucleoside transport across the cell surface, independent laboratories have now confirmed ENT3 as a predominant organelle transporter with localization reported in the lysosome and mitochondria [35,37,196]. This organelle membrane localization functionally allows ENT3 to regulate intracellular and organelle nucleoside levels that are involved in multiple signaling and metabolic processes. Because of this, organelle nucleoside transport (i.e., ENT3) may have profound roles in cellular homeostasis. Consistently, ENT3 is widely expressed in many tissues including bone marrow, liver, placenta, pancreas, skin, heart, GIT, brain, fat, and skeletal muscle, mirroring the pattern of dysfunction observed in SLC29A3-mutated genetic disorders [37,190,191]. Interestingly, when ENT3 transport was examined for cargo selectivity, it was found to transport nucleosides, nucleobases, and monoamines at an obligatory acidic pH relevant to lysosomal and inner mitochondrial subdomains [37]. It was also reported that the ionization states of Asp-219 and Glu-447 in ENT3, which face the opposite sides of the membrane, determined the acidic pH-dependent, transport-permissible, and impermissible states of ENT3 [197,198].

Although ENT3 disorders are monogenic by nature, the pathogenetic relationship of hematopoietic and other dysfunctions observed in SLC29A3 spectrum disorders with ENT3 still remains elusive. Analyses of the SLC29A3 gene on chromosome 10 showed six exons, with mutation sites concentrated in the last exon encoding almost the entire carboxyl half of ENT3 [196]. Characterization of the disease-causing mutations in SLC29A3 found alterations in ENT3 mRNA splicing, protein folding, subcellular trafficking, protein degradation, and pH-sensing ability [196,197,198,199]. Kinetic studies of mutations in ENT3 identified all disease-causing mutations exhibiting moderate to severe reductions in intrinsic transport activities (decrease in V_max_/K_m_) for adenosine [196]. These findings revealed a correlation between human disease severity and residual ENT3 transport activity because mutations with either a complete or partial loss of function tend to exhibit severe or hypomorphic features in humans, respectively [199]. In 2012, the crucial role of ENT3 in the lysosomal homeostasis of macrophages was demonstrated [200]. In 2018, it was subsequently demonstrated that cellular metabolism was significantly altered in peripheral T cells in the absence of ENT3 [201]. Impaired macrophages and T cells account for some pathology in ENT3 disorders (e.g., histiocytosis and autoimmunity); however, the mechanisms of most disease manifestations noted in ENT3 disorders (e.g., abnormalities in other blood cells) remain unknown. By evaluating a gene-trap knockout mouse model for Slc29a3 (encoding mouse ENT3), an indispensable role for ENT3 in the self-renewal and differentiation capabilities of multipotent hematopoietic (HSC) and mesenchymal (MSC) stem cells was later reported. The loss of ENT3 in mice resulted in stem cell exhaustion with myelosuppression, erythroid-myeloid skewing, inflammasome signaling, and breaches of mesodermal tissue integrity, providing the initial clues that damage to nucleoside transporter function in organelles can result in hematological abnormalities. These studies indicate that ENT3 intricately regulates autophagy and lysosomal functions in hematopoietic and mesenchymal stem cells, which partially involve adenosine-dependent AMP-activated protein kinase (AMPK)/ mammalian target of rapamycin (mTOR) signaling. The mouse phenotypes recapitulate the disease symptoms observed in human ENT3 disorders, including anemia, suggesting that Slc29a3-deleted mice are a suitable model for further evaluating ENT3 hematopoietic disease pathologies [190].

Despite significant advances in ENT3 regulation of adult stem cell and blood tissue homeostasis, unfortunately, the loss of ENT3 transport of nucleosides alone could not fully explain the hematological pathologies in Slc29a3 KO mice. Moreover, HSC transplantation in Slc29a3-null mice increased survivability with notable improvement in health, whereas treatment with synthetic adenosine analogs (e.g., AICAR) resulted in only a partial recovery of ENT3 dysfunctions, including anemia, in Slc29a3 KO mice [190]. Transplantation of HSCs may be useful for treating ENT3 disorders; however, further studies are warranted to precisely understand the role of ENT3 in hematological toxicities and the molecular basis of dysfunctions in ENT3 spectrum disorders. Intriguingly, a recent study evaluating Slc29a3 KO mice investigated whether ENT3 transports cargo(s) other than nucleosides that could support adult stem cell health. Through unbiased mass spectrometry-based metabolomic analyses of ENT3-null tissues, this study discovered several bile acids (BAs) as low-affinity cargos of ENT3 that support the ER chemical chaperone function and thereby regulate ER stress signaling indispensable for HSC homeostasis [191]. Indeed, HSCs in Slc29a3 KO mice display high levels of basal ER stress with reduced accumulation of taurocholic acid (a chemical chaperone), suggesting that a lack of utilization of BA contributes to increased ER stress-induced anemia in SLC29A3 disorders [191]. Moreover, many of the abnormalities seen in Slc29a3 KO mice are like those seen in mice with defects in unfolded protein response (UPR) signaling [201,202]. The importance of BA-regulated ER stress signaling in HSCs is further supported by seminal works from the Miharada lab which demonstrated that fetal HSCs exhibit low levels of adaptive responses to ER stress that significantly depend on BA chaperone activity for expansion and differentiation [203,204]. As such, the involvement of ER stress signaling in HSC homeostasis is gaining momentum in research, as seen in several new reports on this topic [205,206,207,208,209]. Importantly, the mechanism that allows ENT3 to regulate erythroid pool size introduces a new concept into the understanding of stress-adaptive ER stress signaling in HSCs [207]. More intriguingly, the ENT3 loss-induced defects in ER stress signaling are not only evident in HSCs but were also observed along the erythroid line of differentiation including hematopoietic progenitors and precursors, which are major targets of anticancer nucleoside analogs. Studying the ENT3 regulation of BA-mediated ER stress signaling in hematopoietic progenitors and precursors constitutes an important area of investigation both from a fundamental science point of view to understand hematopoiesis, and from a translational science perspective to treat anemia in scenarios such as SLC29A3 genetic disorders and nucleoside analog-induced hematological toxicities.

Recently, Mikdar and colleagues investigated the impact of the loss of a plasma membrane ENT member (i.e., ENT1) on human and mouse erythropoiesis and HSC homeostasis [210]. This study demonstrated that ENT1 deficiency in mammals leads to anemia and macrocytosis in mice in the absence of any drug treatments. In addition, this study showed that humans with ENT1 deficiencies manifest reduced human erythrocyte precursor numbers. Using shRNA-mediated knockdown of ENT1 and mice carrying a mutant null allele for Slc29a1 (which encodes ENT1), these authors demonstrated that defective erythropoiesis of hematopoietic progenitors was associated with increased myeloid skewing and extensive dyshomeostasis. Intriguingly, ENT1-mediated adenosine transport was found to be critical for cAMP homeostasis and the regulation of erythroid transcription factors, which contribute to hematopoietic dyshomeostasis. In addition, this study showed that deficiency in ENT1 in human red cells from three patients with the rare Augustine-null [At(a-)] blood type is associated with abnormal red cell morphology, an abnormal nucleotide metabolome, and deregulated protein phosphorylation. In vitro studies on CD34^+^ cells from the patients showed reduced erythroid differentiation in one of the three patients, whereas the other two patients had a concomitant ABCC4 variant encoding the multidrug resistance protein 4 (MRP4) that functions in conjunction with ENT1 to handle adenosine metabolites. The authors also investigated the differences in the erythroid differentiation potential of CD34^+^ progenitors, which was additionally impaired in that patient. Nevertheless, the authors went on to investigate the impact of ENT1 loss on human cells (using shRNA knockdown) and a Slc29a1−/− mouse model which together support the idea of a role for ENT1 in erythropoiesis. The findings were further supported using primary cells from affected individuals and the use of complementary in vitro models in human cells, in addition to a mouse model. However, the relevance of ENT1 for normal erythropoiesis in humans remains to be investigated further, given that the Augustine null blood type has such a mild phenotype. The amelioration of that phenotype by the coinherited ABCC4 variant and the enhancing effect of the ABCC4 inhibitor MK-571 on erythroid differentiation of normal peripheral blood CD34+ cells are intriguing findings; however, questions remain as to why the ABCC4 variants inherited by patients can explain their even milder phenotype. Finally, it is not clear what HSPC types and stages of erythroid differentiation are dependent on ENT1.

## 6. Involvement of Nucleoside Transporters in the Hematopoietic Toxicity of Anticancer Nucleoside Analogs

Anticancer drugs should ideally target cancer cells while avoiding normal cells, including cells in the bone marrow, but this is often difficult to accomplish and remains a major challenge in developing effective therapies. It is possible that anticancer nucleoside drugs can affect mature blood cells or their precursors or progenitors all the way up to HSCs. However, regarding hematopoietic toxicity, the function of the progenitors is far more important, as HSCs are mostly quiescent and often unaffected by the drugs. This is one major reason why leukemic cancer cells can be difficult to treat; progenitors are reached but HSCs (or leukemia stem cells) escape the treatment effect. Whether crosstalk exists between HSC involvement and hematopoietic progenitor regeneration after drug-induced myelosuppression remains less clear. Numerous correlative studies have associated the role of nucleoside transporters with nucleoside analog toxicity on hematopoietic cells; however, evidence for whether nucleoside transporters are a direct determinant of hematological toxicities remains vague. A key likelihood is that, because nucleoside transporters (NTs) are important for the cellular uptake of nucleosides and their corresponding anticancer analog drugs of this trait, they may also be involved in antimetabolite drug sensitivity in off-target sites, perhaps dysregulating the absorption of endogenous nucleosides [211]. Therefore, NTs can be a significant determinant for the toxicity of treatments for various cancers, such as gemcitabine chemotherapy, which is at least partially dependent on sodium-independent ENTs that are now known to play a role in hematopoiesis [212].

In a recent study examining gene–gene interactions of gemcitabine metabolizing enzymes, it was hypothesized that 13 single-nucleotide polymorphisms (SNPs) in the metabolic pathway genes MTHFR and WEE1 are related to gemcitabine-induced hematological toxicities. A total of 132 Chinese patients were evaluated, with 66.7% being males, 57.6% of these with pancreatic cancer, and 81.8% of the patients treated with gemcitabine-based combination chemotherapy. Hematological toxicity was the most common adverse drug reaction (ADR) (92.4%), with neutropenia being the most common (73.5%) and leukopenia being the second most common (71.2%). Interestingly, it was discovered that SNPs rs4877831 and rs7867504 in hCNT3 were heavily correlated with early severe overall hematological toxicity, and rs4877831 and rs7867504 in hCNT3 and rs760370 in hENT1 were heavily correlated with late severe leukopenia. Based on these findings, it was suggested that hENT1 and hCNT3 could even serve as valuable biomarkers for hematological toxicities. The study also concluded that there was evidence to suggest that the gene–gene interaction effects of gemcitabine metabolic genes and WEE1 polymorphisms are related to severe hematological toxicity. It adds that a small sample size and lack of ethnic diversity may have skewed results, so further, larger studies should be conducted to verify the findings. Their focus should be placed on identifying genes and interactions that can diagnose high-risk individuals and prevent ADRs.

In another study examining the correlation of nucleoside and nucleobase transporter gene expression with antimetabolite drug cytotoxicity, new clues were identified to determine whether NTs are related to antimetabolite sensitivity and resistance [211]. The relationship between the expression of nucleoside and nucleobase transporters and antimetabolite cytotoxicity was measured, but it was concluded that the expression of individual transporters may not be a significant factor in cytotoxicity. Although no direct link between transporter expression and cytotoxicity was implicated, there was an increase in sensitivity together with an increase in transporter gene expression between ENT2 and hydroxyurea and CNT1 and O6-methylguanine. P53 status also influenced the relationship between ENT1 gene RNA levels and sensitivity to tiazofurin, AZQ, and 3-deazauridine, of which 3-deazauridine showed a physical interaction with the transporter by inhibiting uridine uptake. Studying the effect of nucleoside transport inhibitors on thymidine salvage and the toxicity of nucleoside analogs in mouse bone marrow granulocyte-macrophage progenitor cells provided further insights. To discover the types of NT present in bone marrow cells, experiments were conducted to determine how NT inhibitors may influence the toxicity of nucleoside analogs and the salvage of thymidine. It was found that the NBMPR-sensitive equilibrative carrier es (i.e., ENT1) is the major nucleoside transporter when NBMPR can prevent damage to the cell from the toxicity of tubercidin. However, further experiments discovered the presence of a CNT that can transport 2-chlorodeoxyadenosine when es is blocked by NMBPR [213].

In addition to ENT1, ENT2 has been implicated in the hematopoietic toxicity of anticancer drugs. Retroviral transfer of the hENT2 nucleoside transporter cDNA conferred broad-spectrum antifolate resistance in mouse bone marrow cells. It was suspected that a method of resistance utilized by tumor cells against antifolate drugs was their nucleoside transporters’ ability to salvage extracellular nucleosides, thereby skipping the requirement of synthesizing nucleotides, which was the biological mechanism targeted by anti-folate drugs. Although research has been performed on specific transporters and their inhibitors, little is known about whether these inhibitors can sensitize tumor cells to antifolates. When tested, it was found that cells that contained the equilibrative NBMPR-sensitive (es) nucleoside transporter (i.e., ENT1) treated with LY231514, a multitargeting antifolate drug, were unaffected even at doses of 1000 nmol/L, but when tested with 1.0 µmol/L and 35 nmol/L NMBPR, the number of colonies dropped below 50% [214]. When myeloid progenitor cells were modified with the equilibrative NBMPR-insensitive (ei) nucleoside transporter gene (i.e., ENT2), which could be expressed through the retroviral transfer of the MeiIRG vector, they remained unaffected by antifolate drugs even when combined with NMBPR. From this, it was determined that es is important in determining toxicity in immature hematopoietic cells, whereas ei is resistant, which may mean that these cells can be preserved by salvage-produced protection through the transfer of MeiIRG. The ei gene is present in many human tissues and will transport all four major nucleosides, which will prevent the cell from receiving toxic levels of nucleosides when ei is overexpressed. This means that chemotherapy can be conducted with a variety of drugs without requiring bone marrow cells to be protected with transduction.

Both purine and pyrimidine nucleoside analogs have been essential parts of leukemia treatments, with cytarabine and fludarabine being the main drugs for the two types of leukemia. Intriguingly, a recent study demonstrated that bone marrow stromal cells can modulate ENT1 activity to protect leukemia cells from cytarabine-induced cell death in mice [215]. Cytarabine-resistant leukemia cells were found to be moderately sensitive to clofarabine in vitro. Clofarabine was approved by the FDA in 2004 and uses hENT1, hENT2, and hCNT3 [171]. Conversely, cytarabine is only known to utilize hENT1, so it was hypothesized that, when leukemia cells acquire resistance against cytarabine, they would become sensitive to clofarabine due to differences in activation pathways. When the 50% growth-inhibitory concentrations (IC_50_) of the two were compared with a leukemia cell line with a 20-fold resistance to cytarabine (HL/ara-C20), it was discovered that clofarabine had only a six-fold resistance, and protein expression analyses found that hCNT1 and deoxycytidine kinase (dCK) expression levels were reduced in HL/ara-C20 when compared to normal human myeloid leukemia cells (HL-60). Overall, clofarabine induced greater apoptosis in both HL-60 and HL/ara-C20 cells than cytarabine. Based on these findings, it was also concluded that clofarabine may be a candidate for second-line treatment for acute myeloid leukemia after cytarabine-based chemotherapy in the case of relapse. Since HL-60 cells are promyeoloblasts isolated from the peripheral blood by leukopheresis from a patient with acute promyelocytic leukemia, the expression status of nucleoside transporters thus becomes an important factor in determining hematological toxicities.

A phase II study was conducted on the effectiveness of clofarabine and cytarabine in adults with relapsed/refractory ALL while also examining whether connective tissue growth factor (CTGF) and nucleoside transporter expression affect the response to these therapies. It was found that there was high expression of various nucleoside transporters in the patients: 54% for hENT1, 44% for hCNT3, 33% for dCK cytoplasmic, and 33% for dCK nuclear. However, it should be noted that the sample size was low, as only 13 subjects were assessed for hENT1 expression and nine for dCK and hCNT3 expression. Overall, the response rate to clofarabine and cytarabine was 17%, and it was found that the complete remission rate between high and low expression of nucleoside transporters did not have a notable difference. Despite this, the study hypothesized that nucleoside transporter expression affects cytotoxicity and still warrants further investigation due to other studies finding correlations between nucleoside transporter expression and tumors; for example, it was found that expression of hENT2 contributes to ex vivo sensitivity to fludarabine [144]. Based on the low response rate, it was decided that a clofarabine/cytarabine regimen is no longer promising enough to justify testing, that patient NT profiles can aid with the use of nucleoside analogs, and that there is potential in drugs that modulate NT and CTGF expression.

As mentioned earlier, gemcitabine has been a mainline treatment agent for pancreatic cancer and is also listed as a possible inclusion in chemotherapies in other cancers [216]. However, the use of gemcitabine is heavily reliant on heavy doses and regular infusions to maintain its effects, a strategy that regularly results in myelosuppression and resistance to gemcitabine [25]. Alternatively, insufficient exposure of gemcitabine to tumor regions due to extensive stromal reactions in pancreatic ductal adenocarcinomas often leads to drug resistance. One of the main ways cells acquire resistance is through dysfunction of nucleoside transporters (NTs) on the cell membrane [217]. To address this issue, the gemcitabine–cardiolipin conjugate (NEO6002) was studied and compared to gemcitabine to test its effectiveness. It was found that NEO6002 is also dose-dependent, and a number of notable improvements in cytotoxicity include faster activity, where NEO6002 displayed its effects after 5 h while gemcitabine required 24 h, and the finding that NEO6002 was able to induce cytotoxicity at higher concentrations than gemcitabine, which was cytostatic after 48 h of incubation. However, the main advantage of NEO6002 over gemcitabine lies in its increased lipophilicity and stability. When tested with cells with nucleoside transporters blocked by NMBPR or dipyridamole, gemcitabine displayed significantly decreased potency, whereas NEO6002 showed no significant changes in potency, suggesting that it enters the cell through an NT-independent pathway. This suggests that NEO6002 can be used in situations where cancer cells have acquired resistance through a lack of expression in NTs. However, whether the strategy augments (or reduces) hematological toxicities requires further experimentation.

A rare and currently incurable B-cell malignancy called Waldenström macroglobulinemia (WM) is a disease that causes proliferation of plasmacytic lymphocytes that cause a variety of complications, including hyperviscosity and bleeding, due to the production of an immunoglobulin M monoclonal protein [218]. It was recommended that a nucleoside analog plus rituximab would produce a desirable effect [219], and the drug 2-chloro-2′-deoxyadenosine (2-CdA) has shown promise in smaller studies with response rates between 55 and 100%, warranting further investigation [15,220]. Rituximab and subcutaneous 2-CdA combination treatment for patients with Waldenström macroglobulinemia was examined, and the clinical and biologic results of a phase II multicenter study were reported. Of the 24 patients tested, there was a response rate of 89.6%, including seven complete responses (CRs), 16 partial responses (PRs), and three minor responses (MRs). The most common adverse effect of the treatment was hematologic toxicity, as 10 episodes (37%) of grade 3 or worse neutropenia were recorded, but there were no long-lasting incidents of neutropenia. It was found that hCNT1 gene expression is significantly correlated with clinical response, with patients with a CR displaying hCNT1 levels that were one log higher than those with a PR or MR. This suggests that NTs are indeed involved in the response to 2-CdA and that genetic variability is an important factor in the response to nucleoside analog drugs. The study suggests that individual NT profiles can be used to optimize therapies (by increasing efficacy and decreasing toxicity) and that hCNT1 may also be involved in other nucleoside analogs that share the same metabolic pathway. Due to this being the first report of the expression of hCNT1 affecting CR in WM, it is recommended that larger studies be conducted to further explore this possibility and any clinical applications.

## 7. Antiviral Nucleoside Analogs Provide Additional Insights into Nucleoside Transporter Involvement in Hematological Toxicities

In addition to anticancer nucleoside drugs, several antiviral nucleoside drugs that cause hematological toxicities are cargos for nucleoside transporters. One of the most-used drugs that causes dose-limiting anemia is ribavirin. Ribavirin is a prodrug that is typically used for the treatment of chronic hepatitis C viral infections [119], but its dosage is limited by hemolytic anemia, which is caused by the accumulation of the phosphorylated metabolites of ribavirin within RBCs [111]. Due to its polarity, its main mode of transport inside cells is plasma membrane nucleoside transporter 1 (ENT1) [221], which is the only transporter displayed by mature human erythrocytes of four members of the transporter family [112]. ENT1 functions by allowing passage to nucleoside salvage inside the cell [113] and has been linked to ribavirin efficacy [114]. Although ribavirin is believed to be mainly transported through ENT1, this has never been directly proven through research. Therefore, the role of ENT1 in the transport and metabolism of ribavirin by human and wild-type or Slc29a1 (encodes ENT1) KO mouse erythrocytes were examined in detail [115]. To investigate this relationship, either wild-type or Slc29a1 KO mice were used to test ENT1′s effects on ribavirin toxicity, and human erythrocytes with ENT1 inhibited with NBMPR were used for comparison. When Slc29a1 (+/−) and KO (−/−) mice were tested, ribavirin uptake decreased drastically in comparison to Slc29a1 (+/+) mice, and no difference was noted when NBMPR was applied to all three mouse types. Human erythrocytes in the presence of NBMPR were also shown to accumulate radioactivity at a much slower pace than Slc29a1 KO mouse erythrocytes within the first six hours. These results suggest that ENT2 does not play a role in the transport of ribavirin, and instead, ENT1 controls its uptake. It was suspected that nonphosphorylated metabolites may impede the uptake of ribavirin in mice, but after testing with RTCOOH, TCONH2, and TCOOH, there was no indication of inhibition up to 1 mM concentration. When modeling erythrocyte disposition data for mice and humans, it was found that human erythrocytes have greater ENT1 activity than mouse erythrocytes, whereas non-ENT1-mediated transport of ribavirin was approximately seven times greater in mice, which may be indicative of the presence of a non-ENT1 transport pathway in mice that is not present in human cells in 10 µM NBMPR. Based on simulations conducted, ENT1 can be judged to be highly active in ribavirin accumulation during long-term administration in human erythrocytes, and it is predicted that ribavirin toxicity would be greatly decreased in Slc29a1 KO (−/−) mice in comparison to (+/+) mice.

Next, the role of nucleoside transporters in the erythrocyte disposition and oral absorption of ribavirin in the wild-type and Slc29a1 KO (−/−) mice was examined. There is evidence that ENT1 is the rate-limiting step in ribavirin accumulation in erythrocytes when experiments are performed in vitro [114], but whether this is true in vivo is still unknown. Based on previous results, it was predicted that ribavirin metabolite accumulation would be up to 15 times higher in the presence of ENT1 than in its absence. However, it was found that there was not much difference between the plasma concentration-time profiles of Slc29a1 (+/+) and Slc29a1 (−/−) mice, and, although there was a predicted difference of 27-fold in the erythrocyte-to-area under curve (AUC) ratio between (+/+) and (−/−) mice, the results showed only a three-fold difference. After some corrections to the model, it was found that phosphorylated metabolic clearance (CLphosp) may play a larger role than expected in the accumulation ratio in vivo. It was suspected that ENTs and CNTs on the basolateral and brush-border membranes of renal epithelial cells may mediate the secretion and absorption of ribavirin [35,115], but it was found in further experiments that this may not be the case. However, CNT2 and CNT3 in the same area of the cell have been found to be linked to the oral absorption of ribavirin [221]. After further investigation, it was found that Slc29a1 KO (−/−) mice were much less dose-dependent in the saturation of transporters than Slc29a1 WT (+/+) mice, which hints that an absence of ENT1 can have a great effect on absorption. It was also found that the presence of ENT1 can modulate the AUC by 3.8-fold due to CNT saturation, which also implies the importance of CNTs in intestinal absorption. However, ENT1 may still be the rate-limiting step and is predicted to cause ribavirin to accumulate in Slc29a1 KO (−/−) mouse intestinal tissue. In another study, it was hypothesized that, in addition to ENT1, ENT2 may contribute to ribavirin uptake. To target ENT2, hypoxanthine (5 mM) and formycin B (50 µM) were used, as they were shown in previous studies to specifically inhibit ENT2 [116]. However, neither compound inhibited the uptake of ribavirin, which suggests that ENT2 does not play a role in ribavirin transport. This also suggests that ENT1 is still the only transporter that facilitates the entry of ribavirin into liver cells (target cells) and hematological cells (off-target cells). Further testing is required to investigate the role of CNTs in the transport of ribavirin. Overall, the studies with ribavirin clearly added support for the involvement of nucleoside transporters in hematological drug toxicities.

## 8. Summary and Future Perspectives

Studies thus far suggest that among various targets, NTs may be key players involved in both normal mammalian hematopoiesis and the occurrence of hematological toxicities after administration of nucleoside analog drugs. Several studies demonstrate the correlative involvement of NTs in hematological toxicities, but the evidence is scant regarding the involvement of specific transporters in hematological toxicities associated with specific drugs or even specific cell type involvement (progenitor cells, precursor cells, etc.). One of the most intriguing observations is that the mice null for the equilibrative NTs (NT-deficient mice) mirror many of the clinical manifestations of nucleoside drugs causing hematological toxicities. It is thus tempting to speculate that nucleoside drugs may hinder the endogenous nucleoside transport function of ENTs in hematopoietic progenitors and precursors to produce adverse hematological drug reactions. Other findings report the direct toxicity of hematopoietic precursors and progenitors by nucleoside drugs due to dysregulation of the cellular metabolism. Moreover, specific variants (SNPs) in ENTs and CNTs (e.g., CNT3) were found to be associated with not only disease-free survival, but also hematological toxicities in multivariate analysis. Therefore, it is likely that the direct impact of nucleoside drugs on hematopoietic precursors, the indirect impact on the loss of endogenous function of NTs, and the altered functional consequences of nucleoside transporter variants on nucleoside analogs may be significant players in clinically observed hematological toxicities [Figure 2]. Mechanistically, several downstream signaling pathways and targets are involved in mediating the effects of nucleoside transporter-dependent hematological toxicities. For instance, the levels of importance of AMP/mTOR-dependent autophagy signaling, adenosine receptor signaling, hormetic ER stress signaling, and inflammasome signaling are gaining relevance to normal hematopoiesis and hematological adverse drug reactions. Very recently, examination of the GATA transcription factor-regulated group of solute carrier transporters demonstrated a nucleoside transporter-dependent differentiation mechanism in erythrocytes [120]. This analysis revealed more than 50 SLC gene cohorts regulated by two GATA transcription factors (GATA1 and/or GATA2) during erythroid differentiation to determine the small molecular composition of erythroid precursors. Intriguingly, ENT1 promoted erythroblast survival and differentiation, and loss of ENT1 in erythroblasts attenuated erythropoiesis and erythrocyte regeneration in response to acute anemia. This study provides a mechanistic interconnection between transcription factors required for early hematopoietic cells and ENT1-mediated endogenous adenosine transport mechanisms for erythroid progenitor cells to efficiently generate erythrocytes. Theoretically, any loss of ENT1 function can compromise erythrocyte formation abilities.

The involvement of both ENT3 and ENT1 in human hematopoietic development sets a stage for future studies in this direction, particularly in understanding the involvement of these transporters in hematological toxicities of anticancer nucleoside analogs. Thus far, studies suggest ENT3′s role as an organelle transporter that can sense the lysosomal contents in adult hematopoietic stem cells, progenitors, and precursors, relaying the information to core cellular machineries including autophagy signaling, ER stress signaling, and nutrient sensing to ensure normal hematopoietic development [190,191]. These findings also suggest that the lysosomal retrograde transport of cargos is critical for the regulation of ER stress during mouse hematopoiesis. Deletion of the ENT3 gene in mice decreases the HSC pool and regenerative capacity, increases myeloid differentiation, and amplifies inflammatory signaling [190,200,201]. Clinical studies suggest the potential association of nucleoside transporters with hematological toxicities, although mechanistic interrelationships for these occurrences are unavailable. If clinically administered nucleoside analogs reach a critical concentration inside target cells that can impair endogenous ENT3 function, then it is conceivable that hematological toxicities can ensue, such as those seen with the ENT3 spectrum of disorders. In addition, ENT1-mediated adenosine transport was found to be critical for cAMP homeostasis and the regulation of erythroid transcription factors essential for hematopoietic homeostasis. Similarly, extracellular concentrations of nucleoside analogs can interfere with endogenous ENT1-mediated adenosine transport to disrupt cAMP homeostasis to precipitate hematological toxicities. Further studies are expected to seek a deep and thorough understanding of the ENT-mediated mechanisms underlying signaling in HSCs to better explain the hematological abnormalities and anemia manifested in nucleoside analog-treated patients. Evaluating the combinations of endogenous and synthetic modulators of nucleoside analogs as potential therapeutic avenues for restoring normal hematopoiesis in ENT deficiency would also be of interest. More studies are expected to determine the molecular mechanisms linking ENTs to AMPK signaling, adenosine receptor signaling, adaptive ER stress signaling, and inflammasome signaling in hematopoietic progenitors and precursors. Generation of hematopoietic cell-specific, constitutive, and inducible ENT KO mouse lines and evaluation in steady-state, stressed, and drug-treated states are also expected to address critical questions linking ENTs to hematopoietic disease pathologies: Is the bone marrow niche-mediated regulation of ENTs indispensable for hematopoietic homeostasis? Do hematopoietic defects occur in a cell-intrinsic or cell-extrinsic manner in ENT-null mice? What is the influence of ENT alleviation of the ER stress response in cytoprotective vs. cytotoxic hematopoietic fates? What is the role of ENTs in the dose-dependent occurrence of anemia? What is the involvement of the ENT-dependent cell signaling response in myeloid bias and inflammasome signaling? Addressing these questions is important as they would uncover the molecular mechanisms by which ENTs relay information to the signaling machinery in hematopoietic cells, and help us understand how such processes have gone awry in nucleoside drug treatments. Finally, unlike ENTs, none of the CNT transporters have been examined in detail for hematopoietic toxicities. This is largely due to a lack of KO mouse models (e.g., CNTs) or a lack of other model systems that can be used to examine the role of CNTs in hematopoietic functions. Future studies are also expected to uncover the role of CNTs in hematopoietic homeostasis.

## Figures and Tables

**Table 1:** Endogenous and synthetic nucleoside transport characteristics of CNTs and ENTs.

Concentrative Nucleoside Transporter	Natural Nucleosides/ Drug(s)	Type	Cell System	Km (µmol/L)	Reference
CNT1	Nucleosides	Adenosine	Cell line	8	[14,15,16]
			Oocytes	-	
		Cytidine	Cell line	3.1 11.5 ± 1.1	[17,18]
			Oocytes	34 ± 7 23 ± 2	[19,20]
		Thymidine	Cell line	27 12.2 ± 0.8	[14,15,18]
			Oocytes	27±2	[20]
		Uridine	Cell line	10.7 ± 1.6 14.41 ± 3.33	[18,21]
			Oocytes	42 56 ± 9 22 ± 3 33 ± 2	[19,20,22,23]
	Drugs	5-azacytidine (5-azaCyd)	Cell line	63 ± 6	[24]
		5-fluorouridine	Oocytes	18 ± 3	[22]
		5-fluoro-2′-deoxyuridine	Oocytes	15 ± 2	[22]
		Gemcitabine	Cell line	32	[25]
			Oocytes	17 ± 2	[19]
CNT2	Nucleosides	Adenosine	Cell line	6	[16,26,27]
			Oocytes	8	[28,29]
		Inosine	Cell line	13.7	[26,27]
			Oocytes	28 4	[26,27,28,29]
		Guanosine		8.5	[14,15]
		Uridine	Cell line	21 46 ± 4 21.43 ± 2.82	[16,21,26,27,30]
			Oocytes	116 40 80	[16,21,28,29]
	Drugs	Cladribine	Cell line	46	[21]
		Clofarabine	-	81	[12]
		Didanosine	Cell line	13	[12,21]
		5-fluorouridine	Cell line	43 ± 7	[30]
CNT3	Nucleosides	Adenosine	-	2.4	[14,15,31]
		Cytidine	-	3.5	[31]
		Inosine	-	4.3	[32]
		Guanosine	-	8.5	[31]
		Thymidine	-	10.6	[14,15,31]
		Uridine	-	5.3 6.68 ± 1.50	[27,31]
	Drugs	Gemcitabine	Oocytes	59.7 ± 17.5	[33]
		3-Deazauridine	Oocytes	50.8 ± 9.90	[33]
ENT1	Nucleosides	Adenosine	Cell line	0.04 ± 0.004 (mM)	[34]
		Cytidine	Cell line	0.58 ± 0.11 (mM)	[34]
		Guanosine	Cell line	0.14 ± 0.01 (mM)	[34]
		Inosine	Cell line	0.17 ± 0.02(mM)	[34]
		Thymidine	Cell line	0.30 ± 0.03 (mM)	[34]
		Uridine	Cell line	0.26 ± 0.02 (mM) 240	[34]
	Drugs	Cladribine	-	23	[12]
		Clofarabine	-	114	[12]
		Fludarabine	-	107	[12]
		Gemcitabine	-	160	[12]
ENT2	Nucleosides	Adenosine	Cell line	0.14 ± 0.02 (mM)	[34]
		Cytidine	Cell line	5.61 ± 0.42 (mM)	[34]
		Guanosine	Cell line	NA	[34]
		Inosine	Cell line	0.05 ± 0.006 (mM)	[34]
		Thymidine	Cell line	0.71 ± 0.05 (mM)	[34]
		Uridine	Cell line	0.25 ± 0.04 (mM)	[34]
	Drugs	Clofarabine	-	328	[12]
		Gemcitabine	-	740	[12]
ENT3	Nucleosides	Adenosine	-	1900	[12]
		Uridine	-	2000	[5,35,36]
	Drugs	Gemcitabine	-	-	[37]

**Table 2:** Anticancer nucleoside drugs producing hematopoietic toxicities.

ANEMIA				
Drugs	Used to Treat	Dose	Frequency (%)	Reference
Azacitidine	Myelodysplastic syndromes, Chronic myelomonocytic leukemia, Acute myeloid leukemia	75 mg/m^2^	64.7	[52,53]
Cladribine	Hairy cell leukemia, non-Hodgkin’s lymphoma	0.12 mg/kg	1–19.2	[54,55,56,57]
Clofarabine	Acute lymphoblastic leukemia; Acute myelogenous leukemia	40 mg/m^2^/day	13–55.8	[46,58]
Cytarabine	Acute nonlymphocytic leukemia, Acute lymphocytic leukemia, Chronic myeloid leukemia, Acute myelogenous leukemia	20 mg–1 g/m^2^/day	0–52	[46,49,51]
Decitabine	Myelodysplastic syndromes, Chronic myelomonocytic leukemia	15 mg/m^2^	35.2–38	[52,59,60]
Fludarabine	Chronic lymphocytic leukemia	25–30 mg/m^2^	10–25	[61,62,63]
Gemcitabine	Ovarian cancer, Breast cancer, Pancreatic cancer, non-small cell lung cancer, squamous cell lung cancer	800–1250 mg/m^2^	15.8–59	[32,64,65,66]
Nelarabine	Hairy cell leukemia	1.5 mg/m^2^	37	[67]
**THROMBOCYTOPENIA**				
Azacitidine	Myelodysplastic syndromes; Acute myeloid leukemia	75 mg/m^2^/day	6.6–85	[53,68,69,70,71,72,73]
Capecitabine	Metastatic breast cancer	0.25–3 g/m^2^/day	3–31	[74,75,76,77,78,79]
Cladribine	Chronic lymphocytic leukemia, Non-Hodgkin’s Lymphoma	0.12 mg/m^2^/day	4.9–36	[56,80,81,82,83]
Clofarabine	Acute Myelogenous Leukemia	40 mg/m^2^/day	42–90	[58,84,85,86,87]
Cytarabine	Myeloid leukemia	3 g/m^2^/day	13–87	[47,50]
Decitabine	Myelodysplastic syndromes	15 mg/m^2^/day	28.9–65	[59,60,88,89,90,91,92]
Fludarabine	Chronic lymphocytic leukemia	30 mg/m^2^/day	4.5–42	[93,94,95,96,97]
Gemcitabine	Breast cancer, non-small cell lung cancer	1000–1250 mg/m^2^/day	44.2–74	[65,66,98,99,100]
Nelarabine	T-cell acute lymphoblastic leukemia	1.5 mg/m^2^/day	18–36	[101,102,103,104]
**LYMPHOPENIA**				
Azacitidine	Myelodysplastic syndromes, Acute myeloid leukemia	75–100 mg/m^2^/day	34.6–37	[70,105,106,107]
Capecitabine	Metastatic breast cancer	0.25–3 g/m^2^/day	67–78	[77,108,109,110]
Cladribine	Chronic lymphocytic leukemia	0.12–3.5 mg/m^2^/day	White cell count decreases by 4% 25%	[81,111]
Cytarabine	Myeloid leukemia	50 g/m^2^/day	T-cell count decreases	[112,113,114]
Decitabine	Myelodysplastic syndromes	1–15 mg/m^2^/day	CD8 T-cells population decreases, CD4 T-cells population remains unaffected.	[34,115,116,117,118]
Gemcitabine	Breast cancer	1000 mg/m^2^/day	15	[33,119]
Nelarabine	T-cell acute lymphoblastic leukemia	1.5 mg/m^2^/day	79	[103,120,121]
**NEUTROPENIA**				
Azacitidine	Myelodysplastic syndromes, Acute myeloid leukemia	75 mg/m^2^/day	41–65.7	[53,68,107,122,123]
Capecitabine	Metastatic breast cancer	0.25–3 g/m^2^/day	8–26	[74,124]
Cladribine	Chronic lymphocytic leukemia, non-Hodgkin’s lymphoma	0.12 mg/m^2^/day	11.9–71	[54,56,57,81]
Clofarabine	Acute myelogenous leukemia	2 m–40 mg/m^2^/day	11–55	[46,84,125,126]
Cytarabine	Acute myelogenous leukemia	1–20 mg/m^2^/day	9–85.7	[45,48,49,50]
Decitabine	Myelodysplastic syndromes	15 mg/m^2^/day	32–90	[60,117,127,128]
Fludarabine	Chronic lymphocytic leukemia	30 mg/m^2^/day	8.2–10.5	[93,129,130]
Gemcitabine	Breast cancer, non-small cell lung cancer	1000 mg/m^2^/day	19–48.1	[33,65,131]
Nelarabine	T-cell acute lymphoblastic leukemia	1.5 mg/m^2^/day	37–46	[67,102,132]
**EOSINOPHILIA**				
Azacitidine	Myelodysplastic syndromes	75 mg/m^2^/day	Eosinophilic pneumonia	[133,134]
Cladribine	B-cell chronic lymphocytic leukemia	0.12 mg/m^2^/day	Eosinophilia	[135,136]
Clofarabine	Leukemia	20 mg/m^2^/day	Eosinophilia	[137,138,139]
Fludarabine	Non-Hodgkin’s lymphoma, Chronic lymphocytic leukemia, Follicular non-Hodgkin’s lymphoma	30 mg/m^2^/day	Peak eosinophil count ranging from 0.87 × 10^9^/L to 7.9 × 10^9^/L	[140,141,142]
Gemcitabine	Pancreatic cancer, Gall bladder cancer	1000 mg/m^2^/day	Patients suffer from eosinophilic pneumonia; peripheral blood eosinophil count 90.3%	[143]

## Data Availability

Not applicable.
